# Effect of Tea Polyphenols on the Melt Grafting of Glycidyl Methacrylate onto Polypropylene

**DOI:** 10.3390/polym14235253

**Published:** 2022-12-01

**Authors:** Xin Zheng, Lina He, Guipeng Yu, Yongjin Li

**Affiliations:** 1 College of Chemistry and Chemical Engineering, Central South University, Changsha 410083, China; 2 Key Laboratory of Organosilicon Chemistry and Material Technology, College of Material, Chemistry and Chemical Engineering, Ministry of Education, Hangzhou Normal University, Hangzhou 311121, China

**Keywords:** polypropylene, melt grafting, glycidyl methacrylate, tea polyphenols (C), co-grafting

## Abstract

It is considered to be one of the most effective strategies to prepare functionalized polypropylene (PP) materials via the melt grafting of polar monomers onto PP chains. However, the grafting efficiency of functional monomers is generally low. To achieve a high grafting efficiency, we explored the effect of tea polyphenols (C), which are good free radical scavengers, on the melt grafting of glycidyl methacrylate (GMA) onto PP chains initiated by dicumyl peroxide (DCP). Specifically, 0.5~3 wt% of tea polyphenols (C) were introduced to the PP/DCP/GMA melt blending system. The morphology, melt flow rate (MFR), thermal and mechanical properties of tea polyphenols (C) incorporated PP/DCP/GMA blends were investigated systematically. The results showed that the proper amount of tea polyphenols (C) (0.5~2 wt%) promoted the grafting of GMA. Unexpectedly, the PP backbone suffered from more severe degradation with the addition of tea polyphenols (C). The phenomena were ascribed to the reaction between phenolic hydroxyl groups of tea polyphenols (C) and epoxy groups of grafted GMA, which was revealed by the FTIR results. In addition, according to DSC and the tensile test, the co-grafting of GMA and tea polyphenols (C) improved the crystallization ability, yield strength and Young’s modulus of the PP matrix.

## 1. Introduction

Polypropylene (PP) is one of the most important polymers widely used in packaging, automotive industry, optical fibers and so on [1,2,3,4,5]. Though PP exhibits advantages, such as a good mechanical property, chemical resistance, ease of processing, low cost and so on, the non-polarity still restricts its applications [6,7]. The grafting of polar monomers onto non-polar PP chains via reactive melt blending is considered as an effective strategy to endow the PP matrix with polarity [8,9,10,11,12]. There are kinds of monomers that can be grafted onto PP chains, such as maleic anhydride [10,13,14,15], glycidyl methacrylate (GMA) [7,16,17,18], acrylic acid [19,20,21], vinyl oxazoline [22] and so on. The melt grafting of GMA has received wide attention due to the high reactivity of the epoxy group of GMA toward carboxyl, hydroxyl or amino groups [23].

The mechanism of the melt grafting reaction of PP has been well investigated and understood [10,18,24,25,26]. In brief, the decomposition of the peroxide generates primary radicals at first. Then the primary radicals abstract the tertiary hydrogen atoms from the PP backbone, resulting in PP macro-radicals which may react with monomers. At the same time, the undesired *β* chain scission of the PP macro-radicals may occur, which has a negative influence on the grafting of the monomer and the properties of the PP matrix. To obtain a high grafting degree of GMA, styrene has been added as a comonomer to reduce the *β* chain scission of the PP backbone and to increase the grafting efficiency of GMA during the melt grafting process [25,26]. The mechanism was proposed to be that, styrene preferentially grafted on the PP macro-radicals to form styryl macro-radicals with a high stability, which then initiate the copolymerization of GMA to form branches [26,27]. However, there are still ambiguities concerning the grafting reactions in the comonomer incorporated system [26]. Furthermore, styrene has a low boiling point, compared to the melt processing temperature and volatile property, which shows potential safety and health hazards [28].

Tea polyphenols (C) are biologically active compounds of plant origin which have the desired effects, such as antioxidant, antibacterial, anticancer, and antiradiation properties [29,30,31,32]. Thus, tea polyphenols (C) have attracted great interest in the food and pharmaceutical industries. Liu et al. [33] fabricated polylactic acid/tea polyphenols (C) composite nanofibers. The results showed that the addition of tea polyphenols (C) improved the antioxidant behavior of the nanofibers. The 1,1-diphenyl-2-picrylhydrazyl (DPPH) radical-scavenging capacities of the nanofibers achieved 95.07% ± 10.55%. The strong radical-scavenging activity of tea polyphenols (C) is ascribed to the presence of the phenolic groups which can generate quininine [34]. Furthermore, due to the chemical reactivity of the hydroxyl groups on aromatic rings, the tea polyphenols (C) are capable to form both noncovalent and covalent bonds [35,36,37]. Shin et al. [35] reviewed the use of pyrogallol-containing polyphenols as molecular glues in biomolecular interactions, modifiers in material-independent surface chemistry, and chemical moieties to form covalent linkages in biomaterials. Ren et al. [37] prepared a highly crosslinked ultrahigh-molecular-weight polyethylene with the assistance of polyphenol. The reactive phenolic hydroxyl groups of the tea polyphenol coupled with free radicals and formed extra crosslinking sites. The crosslinking efficiency enhanced remarkably with the increasing tea polyphenol content. In light of the molecular characteristics and the desired properties of tea polyphenols (C), we explored the effect of tea polyphenols (C) on the melt grafting of GMA onto PP initiated by dicumyl peroxide (DCP). The grafting degree of GMA and the degradation of the PP backbone were studied by FTIR and MFR, respectively. Furthermore, the morphology and properties of the tea polyphenols (C) incorporated PP/DCP/GMA system were investigated. At last, the co-grafting mechanism of GMA and tea polyphenols (C) was proposed.

## 2. Materials and Methods

### 2.1. Materials

The base H501 type polypropylene (PP) used in this work was purchased from Sumitomo Chemical Co., Ltd. (Tokyo, Japan). This kind of PP has a melt flow index of 3.0 g/10 min under 230 °C, 2.16 kg. Dicumyl peroxide (DCP) was obtained from Shenzhen Long Li Feng Material Co., Ltd (Shenzhen, China). Glycidyl methacrylate (GMA, ≥97%) was supplied by Shanghai Aladdin Bio-Chem Technology Co., Ltd (Shanghai, China). Tea polyphenols (C) was bought from Nantong Runfeng Petrochemical Co., Ltd (Nantong, China). All materials were used directly as received. Solvents including Xylene and acetone were general purpose reagent grade.

### 2.2. Preparation of the PP/DCP/GMA/C Blends with Different Composite Ratios

PP and tea polyphenols (C) were firstly dried in a vacuum oven at 80 °C for at least 12 h. Then, the PP pellets, 0.5 wt% of the DCP initiator, 0~5 wt% of GMA and 0~3 wt% of tea polyphenols (C) were dry-blended before charging into a Haake PolyLab QC mixer. The melt grafting reaction took place at 190 °C with a constant rotation speed of 50 rpm for 5 min. The codes and corresponding feed ratios of the blends were given in Table 1.

The blend films with 500 µm thickness were prepared by hot-pressing at 200 °C, 12 MPa and cooling down to 30 °C, by cool-pressing. The purification of the blends was carried out, according to the established literature method [18,38]. Specifically, about 1 g of obtained blend was first dissolved in 50 mL of xylene at 135 °C, followed by precipitating with excess acetone. All samples were dissolved and precipitated three times, then the purified samples were isolated by filtration and dried in a vacuum oven at 80 °C, for at least 12 h. Note that the acetone is the good solvent for both GMA and tea polyphenols (C).

### 2.3. Characterizations

The grafting of GMA and tea polyphenols (C) was determined by the Fourier transformed infrared (FTIR) spectroscopy. The infrared spectra of all purified samples were recorded on a Bruker VERTEX 70v spectrometer at the wave-number range of 400–4000 cm^−1^, with a resolution of 2 cm^−1^ and an accumulation of 64 scans.

The morphology of the PP/DCP/GMA/C blends with different composite ratios was observed using a Hitachi S-4800 type field emission scanning electron microscope (FE-SEM). The observation was carried out at an accelerating voltage of 3.0 kV. All samples were fractured in liquid nitrogen followed by drying in a vacuum oven at 80 °C for at least 12 h. The fractured surface was then coated with a thin layer of gold.

The melt flow rate (MFR) of all samples were measured using a GT-7100MIBH type fusion index instrument at 230 °C with a load of 2.16 kg, according to the GB3682-83 standard.

The non-isothermal crystallization and melting behaviors of all samples were characterized using a Q2000 type differential scanning calorimetry (DSC) under a nitrogen atmosphere. The detailed procedure was as follows: about 5 mg of samples was sealed in an aluminium pan; then the samples were firstly melted at 190 °C for 5 min to eliminate the thermal history, followed by cooling to 30 °C at a cooling rate of 10 °C/min to obtain the crystallization curves. The melting curves were recorded by reheating the samples from 30 °C to 190 °C at a cooling rate of 10 °C/min.

The thermostability of all samples were examined on a Q500 type thermogravimetric analyzer (TGA) from 30 °C to 650 °C under a nitrogen atmosphere with a heating rate of 10 °C/min.

The glass transition temperature (*T*_g_) and storage modulus of the samples were measured using a Q-800 type dynamic thermomechanical analyzer (DMA) under nitrogen atmosphere. The samples were cut into 80 mm × 6 mm × 0.5 mm strips using a standard press knife, and tested with a heating rate of 3 °C/min from −60 to 190 °C with the fixed amplitude of 5 μm and the frequency of 5 Hz.

Tensile tests were performed on an INSTRON-5966 universal testing machine at a constant cross-head speed of 10 mm/min. The dumbbell specimens with a gauge length of 20 mm, gauge width of 4 mm and thickness of 2 mm were prepared by an injection molding machine (Haake Minijet Pro) at a barrel temperature of 200 °C and a mold temperature of 80 °C.

## 3. Results

### 3.1. Morphology of the PP/DCP/GMA/C Blends

**Figure 1:**
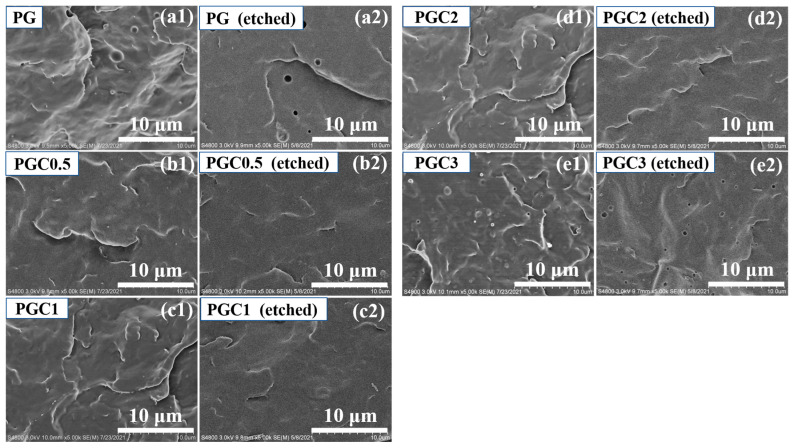
SEM images of the cross-fracture surface of the PP/DCP/GMA/C blends with different tea polyphenols (C) loading before (**a1**–**e1**) and after (**a2**–**e2**) etching.

Figure 1 shows the SEM images of the PP/DCP/GMA/C blends with different composite ratios. Apparent GMA domains were found in PG, which can be etched by acetone. This means the limited grafting of GMA in the PP/DCP/GMA ternary blend. With the addition of 0.5~2 wt% of tea polyphenols (C), as shown in Figure 1b–d, no detectable GMA or tea polyphenols (C) domains were observed. It is expected that the disappearance of the GMA domains was attributed to the enhanced grafting of GMA with the assistance of tea polyphenols (C). However, the domains appeared again with 3 wt% of tea polyphenols (C). Moreover, it can be seen from Figure 1e1 that the morphology of these domains is distinctly different from those in Figure 1a1. Therefore, the domains may be the agglomerates, based on the excess tea polyphenols (C).

### 3.2. MFR of the PP/DCP/GMA/C Blends

**Figure 2:**
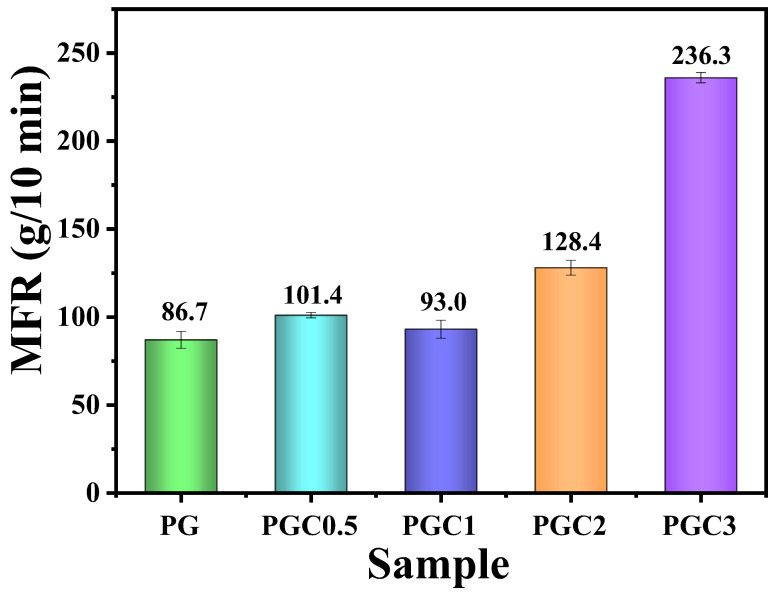
MFR of the PP/DCP/GMA/C blends with different tea polyphenols (C) loading.

The effect of tea polyphenols (C) on the degradation of PP was evaluated by MFR. According to the results shown in Table S1, the MFR of PP-DCP = 100–0.5 was 967 g/10 min. This indicated that the PP matrix suffered from severe degradation when exposed to a high temperature and peroxide. With the incorporation of tea polyphenols (C), the MFR decreased drastically. Especially, PP-DCP-C = 100–0.5–1 has a relatively low MFR of 342 g/10 min. It has been widely reported that the phenolic hydroxyl groups can scavenge free radicals [34]. Therefore, it is reasonable to conclude that tea polyphenols (C) inhibit the degradation of PP by scavenging free radicals in the melt system. Figure 2 exhibited the MFR of the PP/DCP/GMA/C blends with different tea polyphenols (C) loading. The MFR of PG was 86.7 g/10 min, which is much lower than that of the PP-DCP = 50–0.5 blend. It is well known that the PP macro-radicals can undergo two competitive reactions: the grafting of GMA and the β-scission. Therefore, the grafting of GMA would also reduce the β-scission reaction, resulting in a low MFR. The incorporation of tea polyphenols (C) into the PG system was expected to further inhibit the degradation of the PP backbone. However, the PGC blends, especially PGC3 with 3 wt% of tea polyphenols (C), turned out to have a higher MFR, which corresponds to the more severe degradation of the PP backbone, compared to PG. This was due to the reaction between tea polyphenols (C) and GMA, which consumed the phenolic hydroxyl groups in tea polyphenols (C). The reaction mechanism will be discussed later.

### 3.3. Properties of the PP/DCP/GMA/C Blends

#### 3.3.1. Thermal Behavior

**Figure 3:**
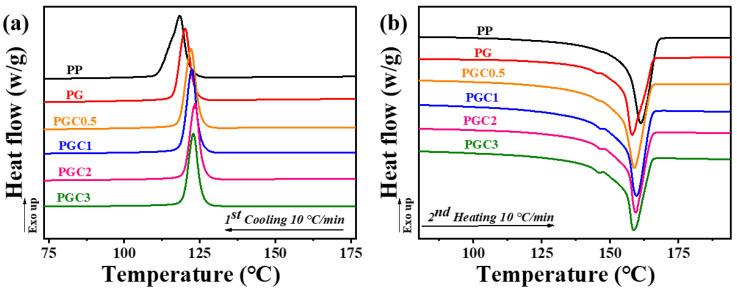
DSC first cooling curves (**a**) and second heating curves (**b**) of PP and the PP/DCP/GMA/C blends with different tea polyphenols (C) loading.

The crystallization and melting behaviors of PP and the PP/DCP/GMA/C blends with different tea polyphenols (C) loading were investigated by DSC. The first cooling and second heating curves were shown in Figure 3. The related parameters, including melting temperature (*T*_m_), crystallization temperature (*T*_c_), melting enthalpy (Δ*H_m_*), and crystallinity (*χ_c_*) were summarized in Table 2. The crystallinity of all samples was calculated by Equation (1) [39] as follows:

(1) $$\chi_{c}=\frac{\Delta H_{m}}{\phi \times \Delta H_{m}^{0}}\times 100\%\;$$

in which the value of $\Delta H_{m}^{0}$ is 209 J/g [39], it means the theoretical melting enthalpy of PP with 100% crystallinity; ϕ means the weight fraction of the matrix in the blends.

According to Figure 3a and Table 2, the semi-crystalline PP has a crystallization temperature of 118.4 °C and a crystallinity of 36.9%. With the grafting of GMA, PG shows a slightly higher crystallization temperature and crystallinity, compared to neat PP. The induction of tea polyphenols (C) further improved the crystallization temperature and crystallinity of the PP matrix. Especially, PGC2 has the highest crystallization temperature among all samples. Moreover, PG and PGCs exhibited a narrower half peak width, compared to PP, which also indicates a better crystallization ability. It is speculated that the enhanced crystallization ability of PG and PGCs was attributed to two main reasons. On the one hand, the *β*-scission in PG and PGCs decreased the molecular weight of the PP backbone, which the increased mobility of the PP molecules; on the other hand, the grafted GMA in PG and the co-grafted GMA-polyphenols in PGCs may act as a heterogeneous nucleation site, which promotes the crystallization of the PP matrix. However, the melting temperature of PG and PGCs was slightly lower than that of PP. In addition, PG and PGCs also show a shoulder peak at about 145 °C. This was because the grafting of GMA and tea polyphenols (C) destroyed the regularity of the PP molecular chains, which induced the defect in the PP crystals and generated the PP crystals with different perfection.

**Figure 4:**
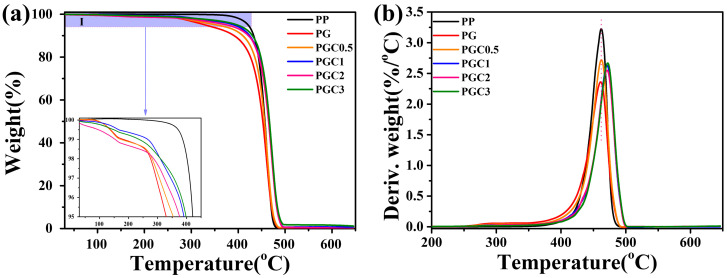
TGA (**a**) and DTG (**b**) curves of PP and the PP/DCP/GMA/C blends with different tea polyphenols (C) loading. The inserted figure at the bottom of (**a**) shows the enlarged I region, which illustrates the initial degradation of the samples.

Figure 4 shows the TGA (a) and DTG (b) curves of PP and the PP/DCP/GMA/C blends with different tea polyphenols (C) loading. It can be seen that PP shows a relatively high initial degradation temperature and takes one step to degrade. On the contrary, PG and PGCs start to degrade at a lower temperature than PP. Moreover, PG and PGCs degrade in multiple steps. This may be due to the *β*-scission of the PP matrix and the degradation of the ungrafted monomers in PG and PGCs. It should be noted that PGCs exhibit an increased thermal stability, compared to PG. The maximum weight-loss peak of PGC1~3 even shifted to a high temperature. Therefore, the combination of tea polyphenols (C) increased the thermal stability of PG. The inherent antioxidant property of tea polyphenols (C) was accounted for the enhanced thermal stability [30,40].

**Figure 5:**
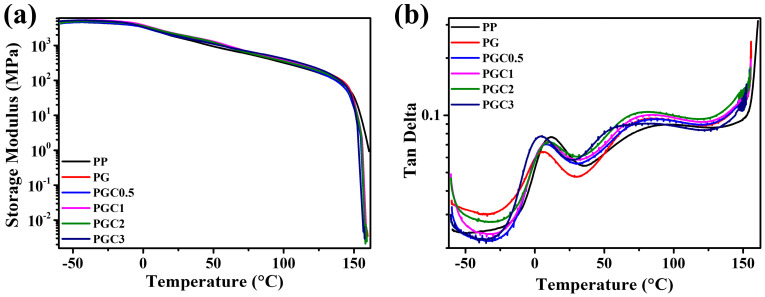
DMA cures of PP and the PP/DCP/GMA/C blends with different tea polyphenols (C) loading: (**a**) storage modulus and (**b**) loss tangent as a function of temperature.

**Figure 6:**
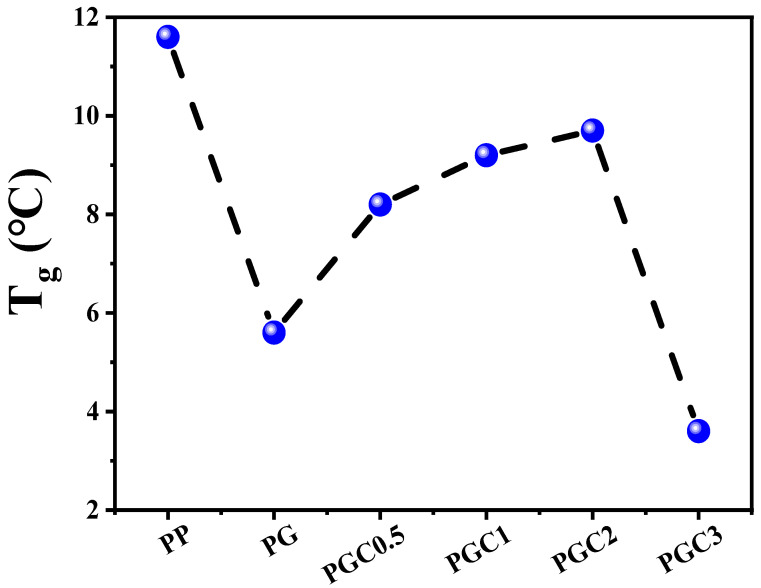
*T*_g_ of PP and the PP/DCP/GMA/C blends with different tea polyphenols (C) loading measured from DMA.

The dynamic mechanical analysis (DMA) was employed to study the glass transition temperature (*T*_g_) of PP and the PP/DCP/GMA/C blends with different tea polyphenols (C) loading. Figure 5 shows the curves of the storage modulus (a) and loss tangent (a) as functions of the temperature measured at a frequency of 5 Hz. The variation of *T*_g,_ according to the different samples, was drawn in Figure 6. As can be seen from Figure 5a, the storage modulus of PP, PG and PGCs decreased gradually at first as the temperature increased, and decreased drastically around the melting temperature. This was a characteristic phenomenon of the semi-crystalline polymers. It can be found from Figure 5b and Figure 6 that PP has a glass transition temperature at 11.8 °C. The considerable degradation of PG and PGCs led to the decrease of the glass transition temperature. However, the glass transition temperature varied a lot, according to the tea polyphenols (C) loading. The glass transition of PG located at 5.8 °C. With the addition of tea polyphenols (C), the glass transition temperature of PGC0.5, PGC1 and PGC2 increased to 7.8 °C, 9.2 °C and 10.4 °C, respectively. This may be due to the co-grafting of GMA and tea polyphenols (C) on the PP chains, which limited the mobility of the PP chain segments. For PGC3 with high tea polyphenols (C) loading, GMA preferred reacting with tea polyphenols (C) to grafting on the PP chains, leading to the consumption of phenolic hydroxyl groups and the reduction of the GMA grafting degree. Consequently, PGC3 has the highest MFR and the lowest glass transition temperature among all samples. The result was in accordance with the SEM and MFR conclusions.

#### 3.3.2. Mechanical Property

**Figure 7:**
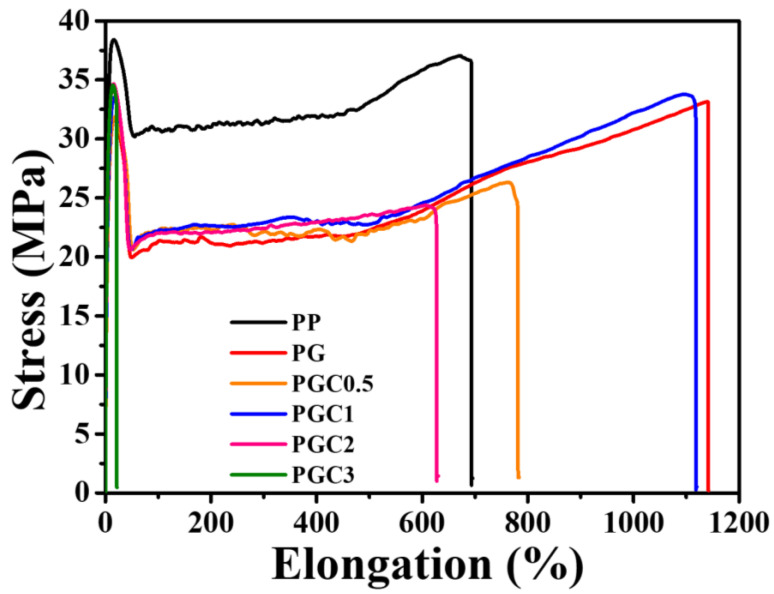
Tensile property of PP and the PP/DCP/GMA/C blends with different tea polyphenols (C) loading.

Tensile tests were performed for PP and the PP/DCP/GMA/C blends with different tea polyphenols (C) loading. Strain-stress curves and related mechanical parameters were shown in Figure 7 and Table 3, respectively. PP shows a high yield strength of 38.6 MPa, a high Young’s modulus of 1173.0 MPa and an elongation of 687.6%. Following the grafting with GMA, the yield strength and Young’s modulus decreased because of the degradation of the PP backbone during the melt grafting. While PGCs showed an improved yield strength and Young’s modulus, compared to PG. In fact, the addition of tea polyphenols (C) did not inhibit the degradation of the PP backbone, according to the MFR results. Thus, the improved mechanical properties were ascribed to the increased crystallinity of PGCs, which was exhibited in Table 2. Notably, PGC3 showed the lowest elongation. This may be due to the obvious agglomerates of tea polyphenols (C) in the PP matrix which induce defects during the tensile process.

### 3.4. Reaction Mechanism

**Figure 8:**
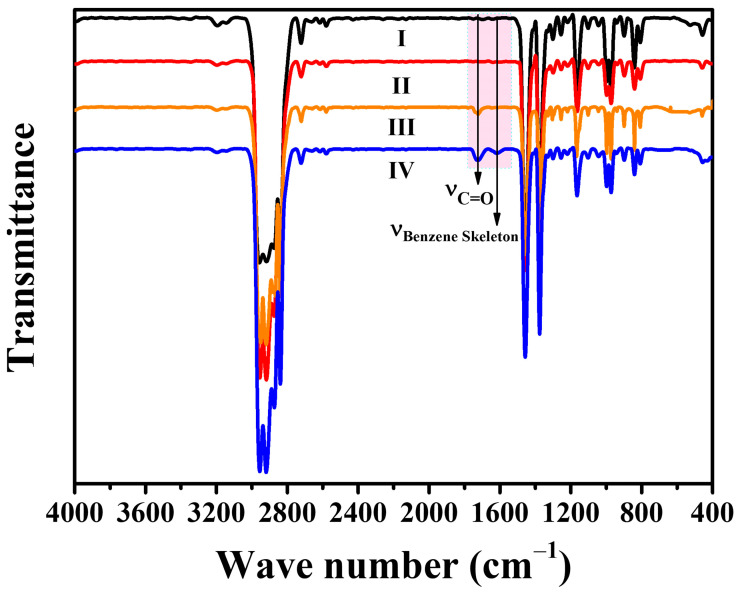
FTIR spectra of PP (I), purified PC1 (II), PG (III) and PGC3 (IV).

**Figure 9:**
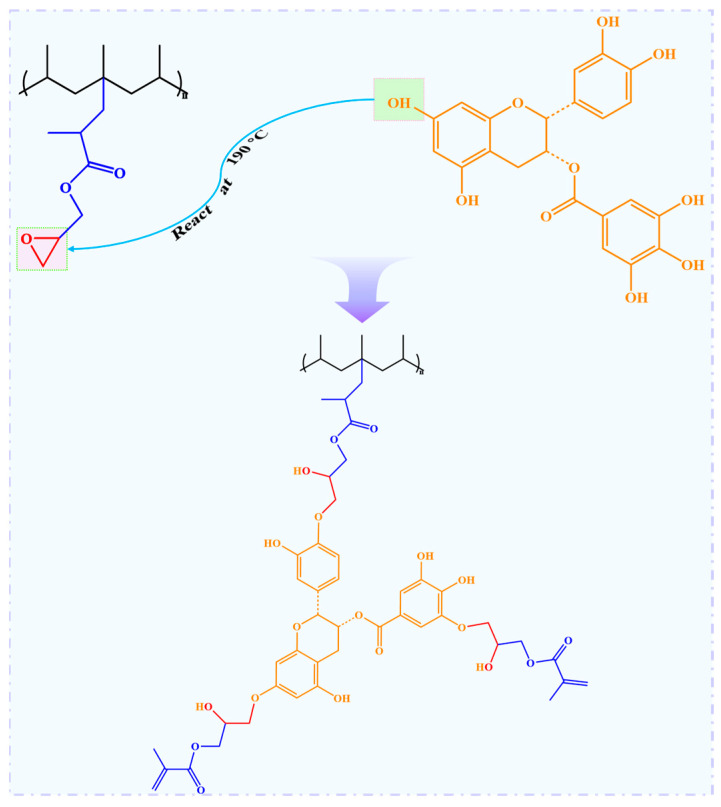
Schematic illustration of the graft reaction of tea polyphenols (C) and GMA onto polypropylene.

**Figure 10:**
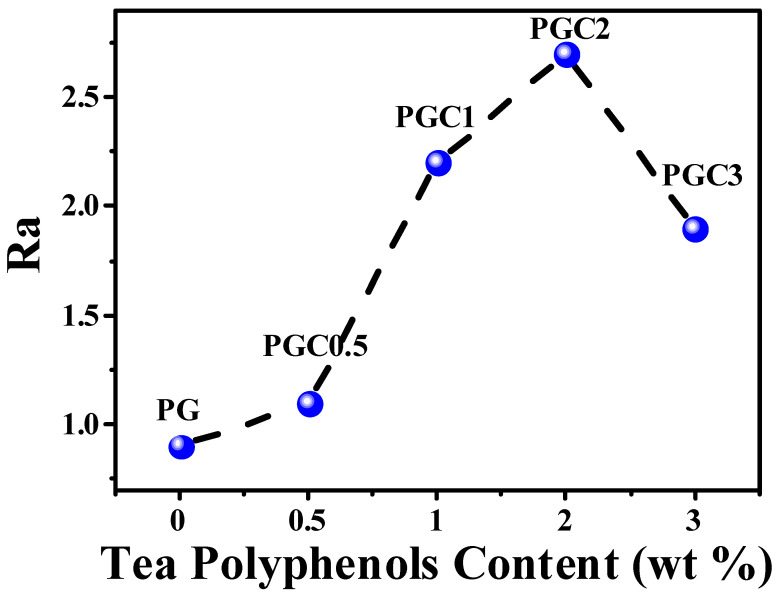
Ra variation of the purified PP/DCP/GMA/C blends with different tea polyphenols (C) loading.

According to the SEM results, 0.5~2 wt% of tea polyphenols (C) facilitate the grafting of GMA. Moreover, tea polyphenols (C) are known as good free radical scavengers. It is expected that the incorporation of tea polyphenols (C) would inhibit the degradation of the PP backbone during the melt-grafting of GMA. However, the PP/DCP/GMA/C blends showed a higher MFR, compared to the PP/DCP/GMA blend. To figure out the reactions during the reactive melt blending, the FTIR spectra of PP, purified PC1, PG and PGC3 were measured using the transmission mode. A comparison of the FTIR spectra of the PP, purified PC1, PG and PGC3 was shown in Figure 8. The spectrum of PC1 is almost the same as neat PP. No detectable bands relating to tea polyphenols (C) were observed. This indicates that tea polyphenols (C) cannot be grafted on the PP chains directly with the initiation of DCP. The characteristic carbonyl stretching vibration band arose at 1722 cm^−1^ in the PG spectrum, which revealed the successful grafting of GMA [34]. It is noteworthy that two bands located at 1726 cm^−1^ and 1617 cm^−1^ were found in the PGC3 spectrum, which were ascribed to the carbonyl stretching vibration band of GMA and the aromatic C = C skeletal vibration of tea polyphenols (C), respectively. As tea polyphenols (C) cannot be grafted on the PP chains directly, it is speculated that the tea polyphenols (C) were grafted by reacting with GMA. In other words, GMA and tea polyphenols (C) were co-grafted on the PP chains. According to the literature, the phenolic hydroxyl groups can react with epoxy groups at a high temperature [41,42]. Therefore, the reaction mechanism was schematically shown in Figure 9. The ratio (Ra = A_1722_/A_2722_) was used to quantify the grafting degree of GMA for PG and PGCs, where A_1722_ and A_2722_ refer to the area of the characteristic carbonyl stretching peak of GMA and the C-CH_3_ stretching peak of the unchanging PP, respectively [18]. The Ra of the purified PG and PGCs with different tea polyphenols (C) loading was shown in Figure 10. Obviously, PG has a relatively low Ra value, implying a low grafting degree of GMA. On the contrary, the Ra value of PGCs is noticeably higher than that of PG. This indicates that the tea polyphenols (C) improve the grafting efficiency of GMA by offering multiple reaction sites and reacting with GMA. Specifically, PGC2 exhibits the highest Ra value. This means 2 wt% is the optimal amount of tea polyphenols (C) for obtaining a high GMA grafting efficiency in this work. The result is consistent with the observation of SEM. The high MFR of PGCs is attributed to the consumption of phenolic hydroxyl groups of tea polyphenols (C) and GMA due to the reactions between GMA and tea polyphenols (C). Especially, PGC3 has a much higher MRF, even compared to PGC2. This is because a high tea polyphenols (C) loading shows a growing tendency to react with GMA, which inhibits the grafting of GMA and in turn inhibits the co-grafting of tea polyphenols (C) onto the PP chains. Consequently, the Ra of PGCs increased at first and then decreased as the tea polyphenols (C) loading increased.

## 4. Conclusions

In this work, we investigated the effect of tea polyphenols (C) on the grafting of GMA, the degradation of the PP backbone via the reactive melt grafting and the properties of the PP matrix. It was concluded that the incorporation of tea polyphenols (C) facilitated the grafting of GMA. With 2 wt% of tea polyphenols (C), the grafting degree of GMA increased three times, compared to PG. The mechanism was proposed to be the reaction between the phenolic hydroxyl groups of tea polyphenols (C) and epoxy groups of GMA during the melt blending. However, the consumption of phenolic hydroxyl groups and GMA monomers also limited their effect on stabilizing the PP macro-radicals, leading to the enhanced degradation of the PP backbone. Despite the obvious degradation, PGCs exhibited potential functionalities originated from the grafted tea polyphenols (C), such as antioxidant, antibacterial and antiradiation properties, which are important for the practical application of the PP product. Moreover, PGCs exhibited an improved crystallization ability, yield strength and Young’s modulus, due to the co-grafting of tea polyphenols (C). This work offered a new path for preparing the grafted PP with a high GMA grafting degree by co-grafting with tea polyphenols (C) during the melt blending process.

## Figures and Tables

**Table 1:** PP/DCP/GMA/C blends with different composite ratios.

Sample	PP (wt%)	DCP (wt%)	GMA (wt%)	Tea Polyphenols (C) (wt%)
PG	100	0.5	5	0
PC1	100	0.5	0	1
PGC0.5	100	0.5	5	0.5
PGC1	100	0.5	5	1
PGC2	100	0.5	5	2
PGC3	100	0.5	5	3

**Table 2:** Thermal parameters measured from the DSC tests for the neat PP and the PP/DCP/GMA/C blends with different tea polyphenols (C) loading.

Sample	*T*_c_ (°C)	*T*_m_ (°C)	$△H_{m}\;(J/g)$	*X_c_* (%)
PP	118.4	161.1	77.0	36.9
PG	120.2	158.2 (145.0)	78.1	39.4
PGC0.5	122.1	158.9 (145.8)	82.2	41.7
PGC1	122.3	159.6 (146.2)	80.6	41.1
PGC2	123.3	159.3 (146.5)	79.1	40.7
PGC3	122.9	158.5 (145.7)	81.7	42.4

**Table 3:** Mechanical parameters of PP and the PP/DCP/GMA/C blends with different tea polyphenols (C) loading obtained from Figure 7.

Sample	Yield Strength (MPa)	Young’s Modulus (MPa)	Elongation (%)
PP	38.6 ± 0.2	1173 ± 7	693.8 ± 77.4
PG	32.1 ± 0.4	891 ± 28	1140.6 ± 65.4
PGC0.5	32.7 ± 0.7	951 ± 33	782.6 ± 102.5
PGC1	33.1 ± 0.4	933 ± 15	1118.0 ± 36.6
PGC2	34.0 ± 0.7	998 ± 23	628.8 ± 37.1
PGC3	34.4 ± 0.1	1050 ± 4	22.1 ± 4.5

## Data Availability

The data presented in this work are available from the corresponding author upon reasonable request.

## Supplementary Materials

The following supporting information can be downloaded at: https://www.mdpi.com/article/10.3390/polym14235253/s1, Table S1: The MFR of the PP-DCP-C blends with different tea polyphenols (C) loading.
