# Supplementary material for: Effect of Tea Polyphenols on the Melt Grafting of Glycidyl Methacrylate onto Polypropylene
Source: Polymers (Basel). 2022 Dec 1;14(23):5253. doi: 10.3390/polym14235253 (PMC9735711; doi:10.3390/polym14235253)
Supplement: Supplementary file 1 [file polymers-14-05253-s001.zip › polymers-2048737-supplementary.pdf]

*Supporting information*

# Effect of Tea polyphenols on the Melt Grafting of Glycidyl Methacrylate onto Polypropylene

**Xin Zheng [1,2], Lina He [2], Guipeng Yu [1,*] and Yongjin Li [2,*]**

[1] College of Chemistry and Chemical Engineering, Central South University, Changsha 410083, Hunan, China
[2] College of Material, Chemistry and Chemical Engineering, Key Laboratory of Organosilicon Chemistry and Material Technology, Ministry of Education, Hangzhou Normal University, Hangzhou 311121, Zhejiang, China
* Correspondence: yongjin-li@hznu.edu.cn (Y. Li).; Tel.: +86-571-2886-7026; gilbertyu@csu.edu.cn (G. Yu); Tel.: +86-731-8883-6961

**Table S1.** The MFR of PP-DCP-C blends with different tea polyphenols (C) loadings.

| Sample | MFR (g/10 min) |
|---|---|
| PP-DCP-C=100-0.5-0 | 967 |
| PP-DCP-C=100-0.5-0.5 | 614 |
| PP-DCP-C=100-0.5-1 | 342 |
